# Monogeneans of West African Cichlid Fish: Evolution and Cophylogenetic Interactions

**DOI:** 10.1371/journal.pone.0037268

**Published:** 2012-05-18

**Authors:** Monika Mendlová, Yves Desdevises, Kristína Civáňová, Antoine Pariselle, Andrea Šimková

**Affiliations:** 1 Department of Botany and Zoology, Faculty of Science, Masaryk University, Brno, Czech Republic; 2 UPMC Univ Paris 06, UMR 7232, BIOM, Observatoire Océanologique, Banyuls/Mer, France; 3 CNRS, UMR 7232, BIOM, Observatoire Océanologique, Banyuls/Mer, France; 4 Institut des Sciences de l'Evolution, IRD-CNRS-UM2, Université Montpellier 2 CC065, Montpellier, France; University of Oxford, United Kingdom

## Abstract

The goals of this paper were to investigate phylogenetic and evolutionary patterns of cichlid fish from West Africa and their *Cichlidogyrus* and *Scutogyrus* monogenean parasites, to uncover the presence of host-parasite cospeciation and to assess the level of morphological adaptation in parasites. This required the following steps, each one representing specific objectives of this paper: (1) to build phylogenetic trees for *Cichlidogyrus* and *Scutogyrus* species based on ribosomal DNA sequences, (2) to investigate phylogenetic relationships within West African cichlid fish based on the analysis of mitochondrial cytochrome *b* DNA sequences, (3) to investigate host-parasite cophylogenetic history to gain clues on parasite speciation process, and (4) to investigate the link between the morphology of the attachment apparatus and parasite phylogeny. Phylogenetic analyses supported the monophyletic origin of the *Cichlidogyrus*/*Scutogyrus* group, and suggested that *Cichlidogyrus* is polyphyletic and that *Scutogyrus* is monophyletic. The phylogeny of Cichlidae supported the separation of mouthbrooders and substrate-brooders and is consistent with the hypothesis that the mouthbrooding behavior of *Oreochromis* and *Sarotherodon* evolved from substrate-brooding behavior. The mapping of morphological characters of the haptor onto the parasite phylogenetic tree suggests that the attachment organ has evolved from a very simple form to a more complex one. The cophylogenetic analyses indicated a significant fit between trees using distance-based tests, but no significant cospeciation signal using tree-based tests, suggesting the presence of parasite duplications and host switches on related host species. This shed some light on the diversification process of *Cichlidogyrus* species parasitizing West African cichlids.

## Introduction

The evolution of African cichlid fish is one of the most dramatic examples of extensive radiation and diversification in animals, reflected in a high number of studies [1]–[4]. However, the recent knowledge on parasitofauna of cichlids is limited to several areas of Africa [5]–[7] and until now, no study of cichlid's parasite evolution has been performed, nor on host-parasite coevolutionary interactions, which could help to understand how parasites have spread and diversified on their cichlid hosts.

Cichlids occur in Africa, Madagascar, Asia and the Neotropics. Their current distribution can be explained by two main hypotheses based on vicariance or dispersal model [8]. The most recent studies seem to favor the vicariance model, but the current knowledge on the distribution and phylogeny (either from morphology or molecules) of cichlids, however, is still not sufficient to eliminate any of the possible scenarios [8]. The monophyly of Cichlidae was assessed using molecular markers [9], [10] or morphological characters [11], [12]. Cichlidae from Madagascar and India form the most basal group of the Cichlidae family and the sister group to the African and Neotropical cichlids [9], [13]. West African cichlids form the most basal African taxa [14].

Among metazoan parasites of cichlids, Monogenea are characterized by high species richness. In general, monogeneans have a direct life cycle and exhibit a high degree of morphological variability and species diversity. Moreover, they are highly host-specific compared to other groups of parasites [15], [16]. They are then a group of choice to study putative morphological adaptation to their hosts, as well as the link between parasite species diversification during their evolutionary history and that of their hosts. The coevolutionary processes in host-monogenean systems have been analyzed previously in numerous studies [17]–[21]. Concerning congeneric monogeneans, host-parasite cospeciation and parasite diversification have been investigated using *Dactylogyrus* gill parasites from freshwater Cyprinidae [21], *Lamellodiscus* gill parasites from marine Sparidae [20], viviparous skin and gill *Gyrodactylus* parasitizing many freshwater and marine fish species [22]–[24] and endoparasitic *Polystoma* parasitizing frogs [25].

**Table 1:** Information about the data sets used for the analyses.

Data set	Number of taxa	Number of characters			Substitution rates						Pi	α	Best fit model
		C	V	P	A-C	A-G	A-T	C-G	C-T	G-T			
SSU+ITS1	29	384	158	99	1.000	2.574	1.000	1.000	4.675	1.000	0.415	0.524	TrNef+I+G
LSU	30	254	279	249	1.000	4.052	1.000	1.000	5.404	1.000	0.299	0.840	TrN+I+G
Cyt *b*	27	188	151	139	0.688	4.908	1.142	0.342	6.786	1.000	0	by codon	GTR+SS (site-specific)

The numbers of conserved (C), variable (V) and parsimony informative (P) characters are shown; Pi – proportion of invariable sites; α – rate heterogeneity approximated by a gamma distribution.

**Figure 1:**
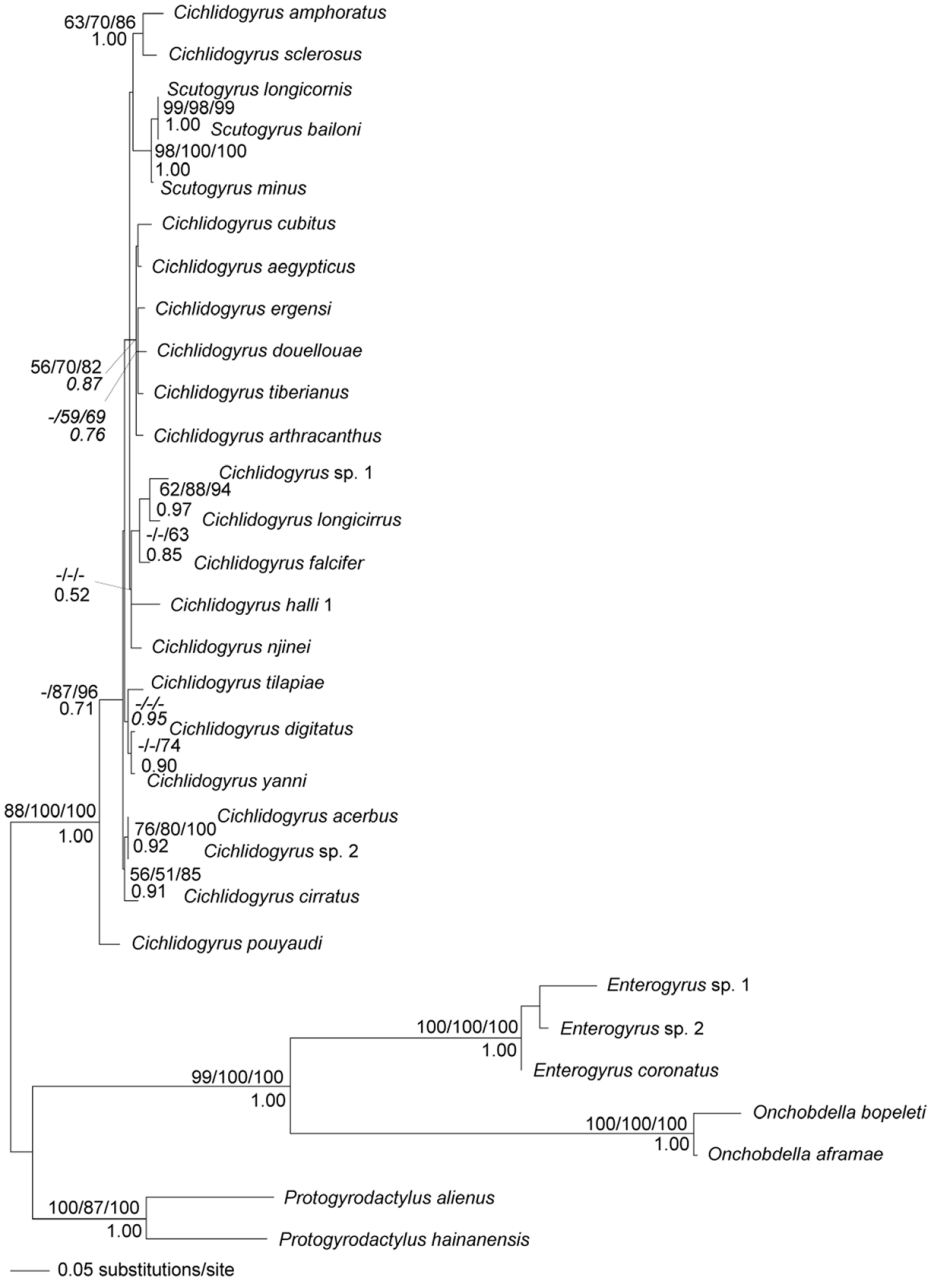
Maximum likelihood tree inferred from analysis of LSU rDNA sequences of parasites. Bootstrap percentages for maximum likelihood, maximum parsimony, minimum evolution (above branches) and posterior probabilities for Bayesian inference (below branches) are shown. Bootstrap values lower than 50 are indicated with dashes.

**Figure 2:**
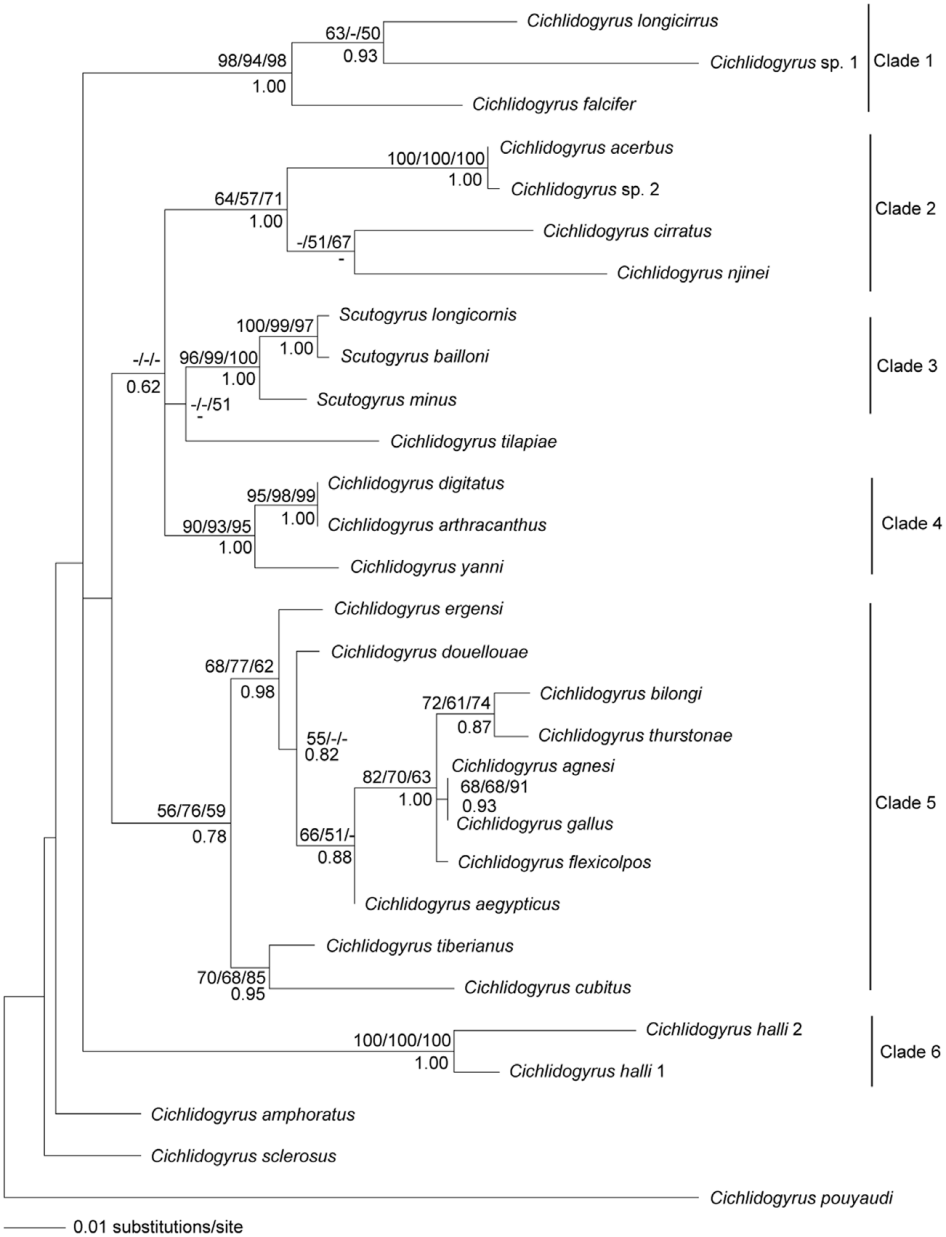
Maximum likelihood tree inferred from analysis of combined partial SSU rDNA and ITS1 sequences of parasites. Bootstrap percentages for maximum likelihood, maximum parsimony, minimum evolution (above branches) and posterior probabilities for Bayesian inference (below branches) are shown. Bootstrap values lower than 50 are indicated with dashes.

**Table 2:** List of monogenean species used in this study, including host species, locality of collection and sequence Accession numbers.

Parasite species	Host species	Locality of collection	SSU and ITS1	LSU
*Cichlidogyrus acerbus* Dossou, 1982	*Sarotherodon galilaeus* (Linnaeus)	Senegal, Africa	HE792780	HQ010036
*Cichlidogyrus aegypticus* Ergens, 1981	*Tilapia guineensis* (Bleeker, 1862)	Senegal, Africa	HE792781	HQ010021
*Cichlidogyrus agnesi* Pariselle & Euzet, 1995	*Tilapia guineensis* (Bleeker, 1862)	Ebrié lagoon, Africa	AJ920286	
*Cichlidogyrus amphoratus* Pariselle & Euzet, 1996	*Tilapia guineensis* (Bleeker, 1862)	Senegal, Africa	HE792782	HE792772
*Cichlidogyrus arthracanthus* Paperna, 1960	*Tilapia guineensis* (Bleeker, 1862)	Senegal, Africa	HE792783	HQ010022
*Cichlidogyrus bilongi* Pariselle & Euzet, 1995	*Tilapia guineensis* (Bleeker, 1862)	Ebrié lagoon, Africa	AJ920287	
*Cichlidogyrus cirratus* Paperna, 1964	*Oreochromis niloticus* (Linnaeus)	Senegal, Africa	HE792784	HE792773
*Cichlidogyrus cubitus* Dossou, 1982	*Tilapia guineensis* (Bleeker, 1862)	Senegal, Africa	HE792785	HQ010037
*Cichlidogyrus digitatus* Dossou, 1982	*Tilapia guineensis* (Bleeker, 1862)	Senegal, Africa	HE792786	HQ010023
*Cichlidogyrus douellouae* Pariselle, Bilong & Euzet, 2003	*Sarotherodon galilaeus* (Linnaeus)	Senegal, Africa	HE792787	HE792774
*Cichlidogyrus ergensi* Dossou, 1982	*Tilapia guineensis* (Bleeker, 1862)	Senegal, Africa	HE792788	HQ010038
*Cichlidogyrus falcifer* Dossou & Birgi, 1984	*Hemichromis fasciatus* Peters, 1857	Senegal, Africa	HE792789	HQ010024
*Cichlidogyrus flexicolpos* Pariselle & Euzet, 1995	*Tilapia guineensis* (Bleeker, 1862)	Ebrié lagoon, Africa	AJ920283	
*Cichlidogyrus gallus* Pariselle & Euzet, 1995	*Tilapia guineensis* (Bleeker, 1862)	Ebrié lagoon, Africa	AJ920285	
*Cichlidogyrus halli* 1 (Price & Kirk, 1967)	*Sarotherodon galilaeus* (Linnaeus)	Senegal, Africa	HE792790	HQ010025
	*Tilapia guineensis* (Bleeker, 1862)	Senegal, Africa		
*Cichlidogyrus halli* 2 Price & Kirk, 1967)	*Oreochromis niloticus* (Linnaeus)	Kossou dam, Africa	AJ920272	
*Cichlidogyrus longicirrus* Paperna, 1965	*Hemichromis fasciatus* Peters, 1857	Senegal, Africa	HE792791	HQ010026
*Cichlidogyrus njinei* Pariselle, Bilong & Euzet, 2003	*Sarotherodon galilaeus* (Linnaeus)	Senegal, Africa	HE792792	HE792775
*Cichlidogyrus pouyaudi* Pariselle & Euzet, 1994	*Tylochromis intermedius* (Boulenger, 1916)	Senegal, Africa	HE792793	HQ010039
*Cichlidogyrus sclerosus* Paperna & Thurston, 1969	*Oreochromis niloticus* (Linnaeus)	Guandong, China	DQ537359	DQ157660
*Cichlidogyrus* sp. 1	*Hemichromis letourneuxi* Sauvage, 1880	Senegal, Africa	HE792794	HQ010027
*Cichlidogyrus* sp. 2	*Sarotherodon galilaeus* (Linnaeus)	Senegal, Africa	HE792795	HQ010028
	*Tilapia guineensis* (Bleeker, 1862)	Senegal, Africa		
*Cichlidogyrus thurstonae* Ergens, 1981	*Oreochromis niloticus* (Linnaeus)	Kossou dam, Africa	AJ920274	
*Cichlidogyrus tiberianus* Paperna, 1960	*Tilapia guineensis* (Bleeker, 1862)	Senegal, Africa	HE792796	HE792776
*Cichlidogyrus tilapiae* Paperna, 1960	*Hemichromis fasciatus* Peters, 1857	Senegal, Africa	HE792797	HQ010029
	*Oreochromis niloticus* (Linnaeus)	Senegal, Africa		
	*Sarotherodon galilaeus* (Linnaeus)	Senegal, Africa		
	*Tilapia guineensis* (Bleeker, 1862)	Senegal, Africa		
*Cichlidogyrus yanni* Pariselle & Euzet, 1996	*Tilapia guineensis* (Bleeker, 1862)	Senegal, Africa	HE792798	HE792777
*Enterogyrus coronatus* Pariselle, Lambert & Euzet, 1995	*Tilapia dageti* Thys van den Audenaerde, 1967	Senegal, Africa		HQ010030
*Enterogyrus* sp. 1	*Sarotherodon galilaeus* (Linnaeus)	Senegal, Africa		HQ010032
*Enterogyrus* sp. 2	*Sarotherodon galilaeus* (Linnaeus)	Senegal, Africa		HQ010031
*Onchobdella aframae* Paperna, 1968	*Hemichromis fasciatus* Peters, 1857	Senegal, Africa		HQ010033
*Onchobdella bopeleti* Bilong Bilong & Euzet, 1995	*Hemichromis fasciatus* Peters, 1857	Senegal, Africa		HQ010034
*Protogyrodactylus alienus* Bychowsky & Nagibina, 1974	*Gerres filamentosus* Cuvier, 1829	Guangdong, China		DQ157650
*Protogyrodactylus hainanensis* Pan, Zhang & Ding, 1995	*Therapon jarbua* (Forsskal)	Guangdong, China		DQ157653
*Scutogyrus bailloni* Pariselle & Euzet, 1995	*Sarotherodon galilaeus* (Linnaeus)	Ivory Coast, Africa	HE792799	HE792778
*Scutogyrus longicornis* Paperna & Thurston, 1969	*Oreochromis niloticus* (Linnaeus)	Senegal, Africa	HE792800	HQ010035
*Scutogyrus minus* Dossou, 1982	*Sarotherodon melanotheron* Rüppel, 1852	Ivory Coast, Africa	HE792801	HE792779

**Figure 3:**
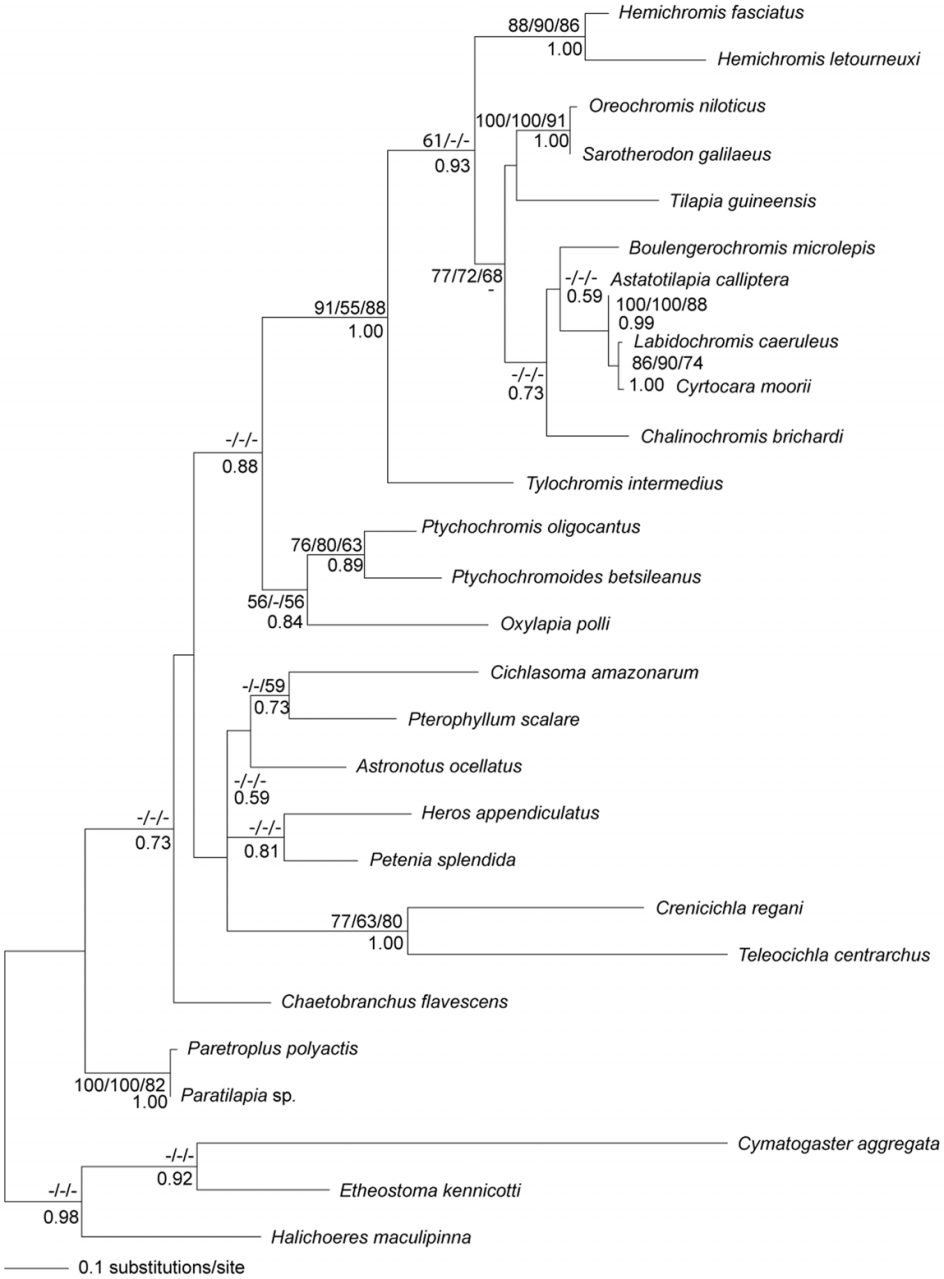
Maximum likelihood tree inferred from analysis of cytochrome *b* sequences of cichlids. Bootstrap percentages for maximum likelihood, maximum parsimony, minimum evolution (above branches) and posterior probabilities for Bayesian inference (below branches) are shown. Bootstrap values lower than 50 are indicated with dashes.

**Figure 4:**
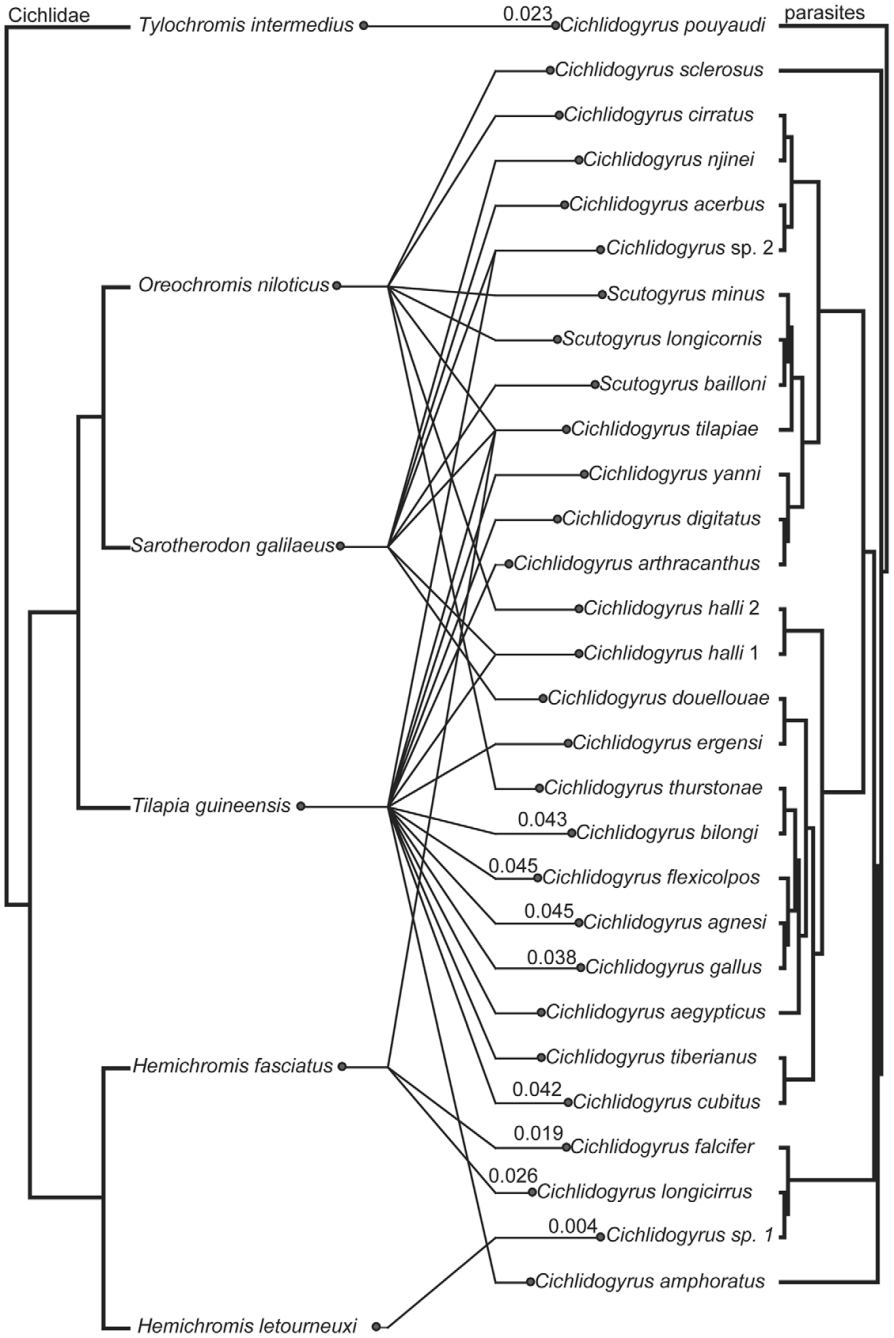
Tanglegram of *Cichlidogyrus/Scutogyrus* species and their hosts. Tanglegram of *Cichlidogyrus*/*Scutogyrus* species and their hosts obtained from comparison of the minimum evolution parasite tree constructed using combined SSU rDNA and ITS1 sequences with the host tree topology resulting from the phylogenetic analyses of cytochrome *b* sequences.

**Table 3:** Results of cophylogenetic analyses with Jane for the cichlid fish and their *Cichlidogyrus* and *Scutogyrus* parasites.

Model	Event costs	Total cost	Cospeciation	Duplication	Host switch	Sorting event	Failure to diverge	P-value
Jane default model	0 1 1 2 1	72	14	42	8	6	10	0.12
TreeMap default model	0 1 1 1 1	66	14	42	8	6	10	0.23
TreeMap default model for building a jungle	0 2 1 1 1	108	14	42	8	6	10	0.40
TreeFitter default model	0 0 2 1 1	26	10	46	3	10	10	**0.01**
Host switch-adjusted TreeFitter model	0 0 1 1 1	23	8	48	7	6	10	0.08
Codivergence adjusted TreeFitter model	1 0 1 1 1	27	0	56	8	9	10	0.08
Equal weights	1 1 1 1 1	79	10	46	7	6	10	0.09

Columns indicate the number of each event type necessary to reconcile host and parasite trees under different event cost schemes. Event costs are for cospeciation, duplication, host switching, sorting event, and failure to diverge, respectively. P-values (in bold when significant) were computed from 999 random reconstructions.

**Figure 5:**
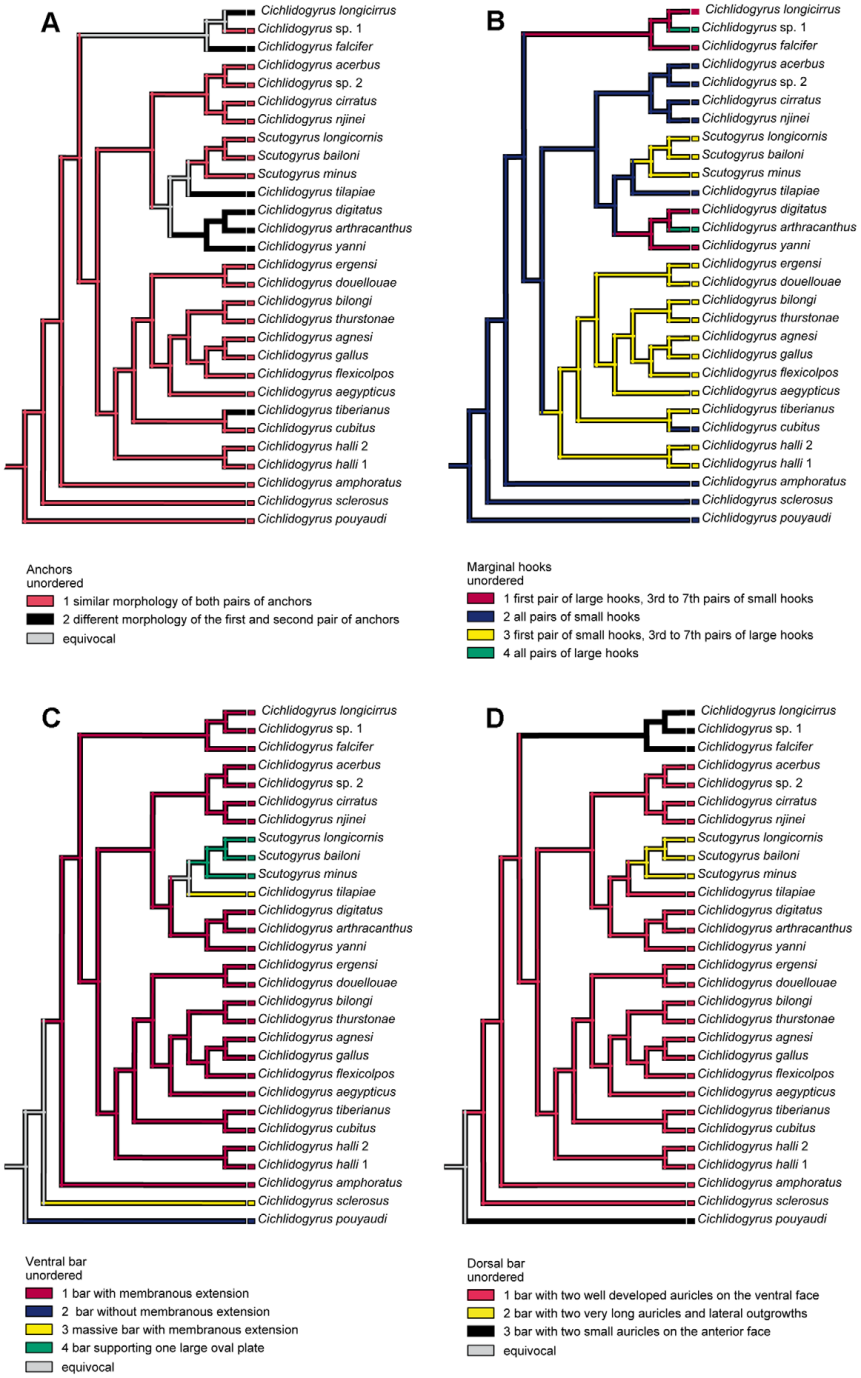
Mapping of haptor morphology onto the minimum evolution parasite tree. (A) shape of anchors, (B) shape of marginal hooks, (C) shape of ventral transverse bar, (D) shape of dorsal transverse bar.

**Table 4:** List of host species used in this study, including locality of collection and sequence Accession numbers.

Host species	Locality of collection	Cytochrome *b*
*Astatotilapia calliptera* (Günther, 1894)	Africa	AF370631
*Astronotus ocellatus* (Agassiz, 1831)	South America	AB018987
*Boulengerochromis microlepis* (Boulenger, 1899)	Africa	AF370632
*Chaetobranchus flavescens* Heckel, 1840	South America	AF370652
*Chalinochromis brichardi* Poll, 1974	Africa	EF679273
*Cichlasoma amazonarum* Kullander, 1983	South America	AF370669
*Crenicichla regani* Ploeg, 1989	South America	AF370646
*Cymatogaster aggregata* Gibbons, 1854	North America	AF370623
*Cyrtocara moori* (Boulenger, 1902)	Africa	AF370634
*Etheostoma kennicotti* (Putnam, 1863)	North America	AF045341
*Halichoeres maculipinna* (Müller & Troschel, 1848)	Western Atlantic	AF370624
*Hemichromis fasciatus* Peters, 1857	Senegal, Africa	HE792802
*Hemichromis letourneuxi* Sauvage, 1880	Senegal, Africa	HE792803
*Heros appendiculatus* (Castelnau, 1855)	South America	AF009951
*Labidochromis caeruleus* Fryer, 1956	Africa	AF370637
*Oreochromis niloticus* (Linnaeus)	Senegal, Africa	HE792804
*Oxylapia polli* Kiener & Maugé, 1966	Africa, Madagascar	AF370626
*Paratilapia sp.* Bleeker, 1868	Madagascar	AF370627
*Petenia splendida* Günther, 1962	Central America	AF370679
*Paretroplus polyactis* Bleeker, 1878	Madagascar	AF370628
*Pterophyllum scalare* (Schultze, 1823)	South America	AF370676
*Ptychochromis oligocantus* (Bleeker, 1868)	Madagascar	AF370630
*Ptychochromoides betsileanus* (Boulenger, 1899)	Madagascar	AF370629
*Sarotherodon galilaeus* (Linnaeus)	Senegal, Africa	HE792805
*Teleocichla centrarchus* Kullander, 1988	South America	AF370647
*Tilapia guineensis* (Bleeker, 1862)	Senegal, Africa	HE792806
*Tylochromis intermedius* (Boulenger, 1916)	Senegal, Africa	HE792807

**Table 5:** Morphological characters of sclerotized parts of the haptor of *Cichlidogyrus* and *Scutogyrus* species (see also Figure 6).

Character 1: Shape of anchors, 2 character states
1.1 similar shape morphology of both pairs of anchors
1.2 different shape of the first (i.e. ventral) pair and second (i.e. dorsal) pair of anchors
Character 2: Shape of marginal hooks, 4 character states
2.1 first pair of large hooks, second pair of small hooks, 3^rd^ to 7^th^ pairs of small hooks
2.2 all pairs of small hooks
2.3 first and second pairs of small hooks, 3^rd^ to 7^th^ pair of large hooks
2 4 first pair of large hooks, second pair of small hooks, 3^rd^ to 7^th^ of large hooks
Character 3: Shape of ventral bar, 4 character states
3.1 bar with membranous extension
3.2 bar without membranous extension
3.3 massive bar with membranous extension
3.4 bar arched, supporting one large, thin, oval plate marked by fan-shaped median thickenings
Character 4: Shape of dorsal bar, 3 character states
4.1 bar with two well-developed auricles attached by a thin foot to the ventral face of the bar
4.2 bar with two very long auricles and lateral outgrowths
4.3 bar with two small, hollow auricles on the anterior convex face

**Figure 6:**
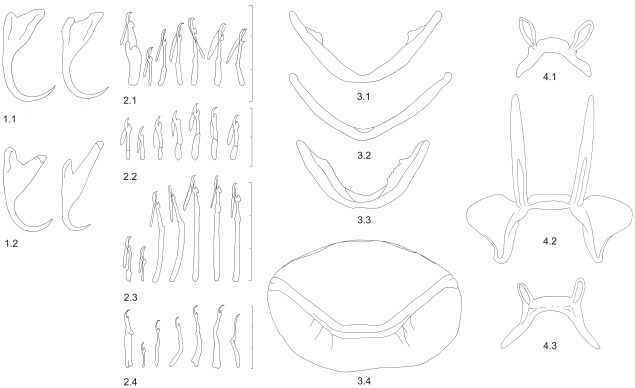
Morphological characters of sclerotized parts of the parasite haptor. Character 1: anchors; character 2: marginal hooks; character 3: ventral bar; character 4: dorsal bar (see Table 5 for description of character states).

African cichlids are parasitized by five genera of monogeneans belonging to the Dactylogyridea, *Cichlidogyrus* Paperna, 1960, *Scutogyrus* Pariselle & Euzet, 1995, *Onchobdella* Paperna, 1968, *Enterogyrus* Paperna, 1963 and *Urogyrus* Bilong-Bilong, Birgi & Euzet, 1994. While *Enterogyrus* and *Urogyrus* are mesoparasitic monogeneans of cichlids, *Cichlidogyrus*, *Scutogyrus* and *Onchobdella* are gill ectoparasites. *Cichlidogyrus* is the most diverse genus of monogeneans parasitizing cichlid fish, which are distributed among a wide range of fish species (more than 40 species within 11 genera) [26]. Both *Scutogyrus* and *Onchobdella* are restricted to several cichlid species; more precisely, *Scutogyrus* is restricted to *Sarotherodon* and *Oreochromis*, and *Onchobdella* to *Hemichromis*, *Chromidotilapia* and *Pelmatochromis*. Following Pariselle and Euzet [5], 71 *Cichlidogyrus* species, 6 *Scutogyrus* species, 8 *Onchobdella* species and 8 *Enterogyrus* species were described in cichlid fish from Africa, the Levant and Madagascar, among them 38 monogenean species (including the genera *Cichlidogyrus*, *Scutogyrus*, *Onchobdella* and *Enterogyrus*) were reported in cichlid fish of West Africa and 22 species were revised in Senegal. Many *Cichlidogyrus* and *Scutogyrus* species are host-specific, i.e. from a total of 54 *Cichlidogyrus* and *Scutogyrus* species infesting West African cichlids, 36 species infest only a single cichlid species and 18 species infest two or more cichlid species [26]. Lateral transfer (i.e. host switch) commonly occurs even between phylogenetically distant cichlid species in artificial and natural conditions [26], [27]. In addition, host switching and parallel speciation processes were hypothesized as the most appropriate evolutionary scenario explaining the repartition of *Cichlidogyrus* groups on *Tilapia*, *Oreochromis* and *Sarotherodon* [26]. However, until now, no cophylogenetic analysis was performed to test this hypothesis.

Monogenean species determination is generally carried out using morphology and size of sclerotized parts of the attachment apparatus (termed haptor) and reproductive organs. Morphological characters have also been used to infer phylogenetic relationships between monogenean species. Concerning *Cichlidogyrus* and *Scutogyrus* species, Pouyaud et al. [26] stated that the morphology of their haptoral sclerites is more suitable for inferring phylogenetic relationships than the morphology of their reproductive organs, which seems to be more useful for resolving species-level identification, presumably because of its faster rate of change. Inter-species variability in the morphology of reproductive organs is in line with the hypothesis of reproductive isolation between phylogenetically related monogeneans facilitating species coexistence within host species [28], [29]. Pouyaud et al. [26] performed phylogenetic analyses based on morphological data (i.e. measurements of haptoral sclerites) and subsequently divided *Cichlidogyrus* and *Scutogyrus* into four groups: “halli”, “scutogyrus”, “tiberianus” and “tilapiae” (this categorization was confirmed by Vignon et al. [30]). However, even if genetic distance based on SSU and LSU rDNA sequence data supports such division in different morphological groups, the molecular phylogenetic trees performed in their study were inconclusive. Pouyaud et al. [26] suggested that *Cichlidogyrus* and *Scutogyrus* parasites can be separated into two groups: parasite species infesting only mouthbrooder cichlids (genera *Oreochromis* and *Sarotherodon*), and species infesting only the substrate brooder cichlids (genus *Tilapia*). Generally, indeed, a given species of *Cichlidogyrus* or *Scutogyrus* does not infect both mouthbrooders and substrate-brooders.

In this paper, we aimed to clarify these points using molecular phylogenetic trees for cichlid fish and their parasites, in order to study the evolution of feeding behavior in fish and morphology in parasites from independent evidence, i.e. molecular data. The objectives of this study were then to perform phylogenetic analyses of *Cichlidogyrus* and *Scutogyrus* species parasitizing cichlid fish in West Africa based on ribosomal DNA sequences, and phylogenetic analyses of Cichlidae to clarify the phylogenetic relationships among the cichlid fish living in West Africa using mitochondrial cytochrome *b* DNA sequences, and to investigate speciation processes in cichlid specific monogeneans using cophylogenetic analyses. In addition, parasite morphological characters were mapped onto parasite phylogeny to study the structural evolution of the haptor that could be related to adaptation to the host, and speciation processes.

## Results

### Parasite phylogeny

The partial LSU rDNA sequences included 20 *Cichlidogyrus* species, 3 *Scutogyrus* species and 7 remaining species as outgroup (*Enterogyrus coronatus*, *E.* sp. 1, *E.* sp. 2, *Onchobdella aframae*, *O. bopeleti*, *Protogyrodactylus alienus* and *P. hainanensis*). LSU sequences of *Cichlidogyrus* and *Scutogyrus* species varied from 637 bp (*C. ergensi*) to 844 bp (*C. arthracanthus*). No variability was observed among the individuals of *C. tilapiae* found in four different host species, i.e. *H. fasciatus*, *O. niloticus*, *S. galilaeus* and *T. guineensis*, and between *Cichlidogyrus* sp. 2 from *S. galilaeus* and *T. guineensis*. However, some nucleotide variability (i.e. p-distance corresponding to 0.037) was found between the individuals of *C. halli 1* found in *S. galilaeus* and *T. guineensis*, and *C. halli 2* found in *O. niloticus*. An unambiguous alignment spanned 533 positions. Information on the LSU rDNA alignment, as well the model selected by ModelTest and its parameters, are shown in Table 1. The MP analysis provided 56 equally parsimonious trees with 669 steps (CI = 0.661, RI = 0.757). All phylogenetic analyses yielded a similar tree topology (Figure 1).

Based on the analyses of LSU rDNA sequences, *Cichlidogyrus* with *Scutogyru*s species (i.e. *Cichlidogyrus*/*Scutogyrus* group) parasitizing Cichlidae formed a strongly supported monophyletic group, with *Cichlidogyrus pouyaudi* in basal position relative to other *Cichlidogyrus* and *Scutogyrus* species, which was strongly supported by bootstrap values (ME and MP analyses) and moderately supported by Bayesian posterior probabilities.

The second data set included the sequences composed of partial SSU rDNA and the entire ITS1 region obtained for *Cichlidogyrus* and *Scutogyrus* species. Partial SSU rDNA sequences varied from 423 bp (*S. bailloni, C. yanni*) to 483 bp (*C. ergensi, C. falcifer, C. tilapiae, S. longicornis*), and ITS1 sequences ranged between 330 bp (*S. bailloni, S. minus*) and 498 bp (*C. pouyaudi*). The partition homogeneity test implemented in PAUP*4b10 was used to test the congruence of the phylogenetic signal in partial SSU rDNA and ITS1 alignment. No significant difference was found (p = 0.1), sequences were combined, and the concatenated alignment spanned 542 positions (see Table 1 for details). Phylogenetic analyses based on partial SSU rDNA and ITS1 sequences included 26 *Cichlidogyrus* species and 3 *Scutogyrus* species. *Cichlidogyrus pouyaudi* was selected for rooting the tree following the results of phylogenetic analyses based on partial LSU rDNA sequences. Information about the data sets used in the analyses of combined SSU rDNA and ITS1 sequences and the model selected by ModelTest are included in Table 1. The MP analysis provided 16 equally parsimonious trees with 367 steps (CI = 0.586, RI = 0.674). ML, MP, ME analyses yielded congruent trees (Figure 2).

All phylogenetic analyses supported the monophyly of *Scutogyrus*, in spite of the slight topological differences between trees; this brings support to the validity of this genus. Overall, six clades of gill monogeneans were recognized using phylogenetic reconstructions based on combined SSU rDNA and ITS1 sequences. Clade 1, strongly supported by all phylogenetic analyses, included three *Cichlidogyrus* species parasitizing *Hemichromis* species i.e. *C. longicirrus*, *Cichlidogyrus* sp.1 and *C. falcifer*. Clade 2, with weak or moderate support values resulting from different phylogenetic analyses, included 4 *Cichlidogyrus* species parasitizing three different cichlid fish species. *Scutogyrus minus, S. longicornis* and *S. bailloni* formed a monophyletic group (clade 3). The position of *Cichlidogyrus tilapiae* parasitizing four different cichlid species (see Table 2) was unresolved. Clade 4, with strong support values, included three *Cichlidogyrus* species parasitizing *Tilapia*. Clades 2, 3 and 4 and *C. tilapiae* formed a weakly supported group using BI analysis. The large clade 5 with weak to good nodal support depending on the phylogenetic method applied (see Material and Methods, part “Phylogenetic analyses of parasite species”, for the definition of nodal support values) was formed by two groups strongly supported by BI; this clade included 10 *Cichlidogyrus* species, among them 8 are parasites of *Tilapia guineensis*. The strongly supported clade 6 included *Cichlidogyrus halli* collected from three different cichlid hosts, *Sarotherodon galilaeus*, *Tilapia guineensis* and *Oreochromis niloticus*.

### Host phylogeny

An unambiguous alignment of cytochrome *b* sequences from cichlids spanned 342 positions. All analyses yielded congruent topologies among the phylogenetic trees. The MP analysis resulted in 2 equally parsimonious trees of 777 steps (CI = 0.319, RI = 0.447). In ML reconstructions, the use of the codon model produced a tree with a low resolution (although congruent with other, more resolved, phylogenetic hypotheses for cichlids obtained here). We then kept the well-resolved tree obtained using the codon partition scheme (Figure 3). Parameters of codon partition model are included in Table 1.

### Cophylogenetic analysis

For parasites, only the tree based on SSU rDNA and ITS1 sequence data (Figure 2) was used because of the much higher clade support values than in the LSU tree (Figure 1). The tanglegram showing associations between *Cichlidogyrus* and *Scutogyrus* monogenean species and their cichlid fish hosts, based on ME phylogenetic trees, is presented on Figure 4. We used two methods to assess the level of cophylogenetic signal in these host-parasite associations: 1. ParaFit, a method which compares patristic distance between host pairs and their corresponding parasites across the whole association and is able to take into account multiple parasites/hosts per hosts/parasites if any, and 2. Jane, a method comparing the two tree topologies (considering branch lengths) that optimally fits the parasite tree onto the host tree by mixing different kinds of coevolutionary events with predefined costs. The optimal fit is found by minimizing the global cost of the reconstruction. In both approaches, the observed structure is tested against a distribution generated from random associations to assess if it is statistically significant, and ParaFit tests the effect of individual host-parasite associations (“links”) on the global fit (see Material and Methods section for additional details and references). Using ParaFit, the overall cophylogenetic structure was significant (with ME (p = 0.001) or ML (p = 0.047) trees). Nine individual links out of 34 contributed significantly to this global fit (see Figure 4) using ME trees, but only 3 with ML trees (*Hemichromis fasciatus* – *Cichlidogyrus longicirrus*, *H. fasciatus* – *C. falcifer*, *H. letourneuxi* – *Cichlidogyrus* sp. 1).

Using different cost schemes in Jane generated different results (Table 3), but the significance of the global cost (P = 0.01) was only attained in the TreeFitter default model (cost settings 0 for cospeciation, 0 for duplication, 2 for host-switch, 1 for loss, 1 for failure to diverge (added to the original TreeFitter cost only based on the four first types of coevolutionary events)). Among the cost sets tested here, this set of costs has the highest host-switching cost. In all reconstruction, the number of duplications (i.e. parasite speciation without host speciation, or intrahost speciation) was very high. All other cost schemes used resulted in a non significant fit between parasite and hosts phylogenies.

### Mapping of the morphology of the attachment apparatus onto the parasite phylogeny

The mapping of anchor morphology suggests that similar shape for both anchor pairs is their ancestral character state, and then that anchors with different shapes represent a derived condition (Figure 5A). The mapping of marginal hooks shape onto the parasite phylogenetic tree (Figure 5B) suggests that having all pairs of small marginal hooks (i.e. 2.2) is the ancestral state in *Cichlidogyru*s. From this ancestral state, two different character states of marginal hooks may have derived (representing character states 2.1 in *Cichlidogyrus* and 2.3 found in *Cichlidogyrus* and *Scutogyrus*). Mapping also suggests the occurrence of two independent changes in the shape of marginal hook toward a state where all pairs of hooks are large (character state 2.4), one in *Cichlidogyrus* sp. 1 parasitizing *Hemichromis letourneuxi* and the other in *Cichlidogyrus arthracanthus* parasitizing *Tilapia guineensis*. The ancestral state of the ventral transverse bar (Figure 5C) could not be hypothesized from this analysis but the morphological type with membranous extension (i.e. character state 3.1) was observed in the majority of species. Two changes in the shape of the ventral transverse bar toward a massive bar with membranous extension (character state 3.3) were inferred in *Cichlidogyrus tilapiae* and *Cichlidogyrus sclerosus*, as well as one change in the ventral transverse bar with membranous extension toward the bar supporting one large oval plate (character state 3.4) in *Scutogyrus* species. We could not identify the ancestral state of the dorsal transverse bar (Figure 5D), but dorsal bars with well developed auricles (character state 4.1) were observed in the majority of *Cichlidogyrus* species. A bar with two small auricles on the anterior face was found in *C. pouyaudi* and *Cichlidogyrus* parasitizing *Hemichromis* species. One change in this state toward the dorsal bar with two long auricles and lateral outgrowths was inferred in *Scutogyrus*.

## Discussion

### Phylogenetic status of gill monogeneans parasitizing cichlid fishes

Based on phylogenetic analyses of LSU rDNA using as outgroup specific gill parasites of *Onchobdella*, endoparasitic *Enterogyrus* (found in *Sarotherodon* and *Tilapia* species) and *Protogyrodactylus* (a parasite genus selected following Mendlová et al. [31], but not included among cichlid parasites), we investigated whether or not the *Cichlidogyrus*/*Scutogyrus* group is monophyletic, and confirmed this was the case. Previously published molecular phylogenetic analyses suggested *Cichlidogyrus* to be a polyphyletic taxon [26], [31], [32] and pointed to the different origins for endoparasitic *Enterogyrus* and ectoparasitic *Onchobdella* (specific to *Hemichromis* species) compared to gill monogeneans *Cichlidogyrus* and *Scutogyrus* [31]. Moreover, the non-monophyletic origin of *Scutogyrus* was supported by phylogenetic analyses of ribosomal DNA sequences [32]. It has been proposed that the *Scutogyrus* genus arose from *Cichlidogyrus*, according to the morphology of dorsal and ventral transverse bars [33]. In the present study, based on the phylogenetic analyses using LSU, SSU rDNA and ITS1 sequences, we suggest that *Scutogyrus* species form a monophyletic group contrary to Wu et al. [32]. However, we confirmed the polyphyletic origin of *Cichlidogyrus*, suggesting the need for a taxonomical revision of this genus.

### *Cichlidogyrus*/*Scutogyrus* phylogeny: a link to behavioral strategies of cichlid fish

The phylogenetic analyses using LSU rDNA sequences performed in this study placed in basal position *Cichlidogyrus pouyaudi* parasitizing *Tylochromis intermedius*, which suggests that this parasite diverged earlier than the other *Cichlidogyrus* and *Scutogyrus* species. *Cichlidogyrus pouyaudi* was originally described from *Tylochromis jentinki* in West Africa [34], where it was observed that the structure of the dorsal transverse bar of this parasite species is different of the other *Cichlidogyrus* species. Pariselle and Euzet [34] suggested that such haptor morphology represents an archaic feature in *Cichlidogyrus* species living on ancient cichlid fish such as *Tylochromis* species.

In this study, six clades of gill parasites within *Cichlidogyrus* and *Scutogyrus* were identified using phylogenetic analyses based on SSU and ITS1. Two clades only contain strictly host specific *Cichlidogyrus* parasites, i.e. clade 1 parasitizing *Hemichromis* species and clade 4 parasitizing *Tilapia guineensis*. Contradictory to the prediction of Pouyaud et al. [26], we found that three *Cichlidogyrus* species parasitized both mouthbrooders and substrate-brooders. Clade 2 included *Cichlidogyrus* parasitizing mouthbrooder cichlids (i.e. *Oreochromis* and *Sarotherodon*), except *Cichlidogyrus* sp. 2 found on the mouthbrooder *Sarotherodon galilaeus* as well as the substrate-brooder *Tilapia guineensis*. The absence of other *Cichlidogyrus* parasitizing *Tilapia guineensis* in this clade suggests a secondary host transfer of *Cichlidogyrus* sp. 2 from mouthbrooders to substrate-brooders. *Cichlidogyrus thurstonae* and *C. douellouae*, both parasites of mouthbrooders (in clade 5 of phylogenetic trees), probably colonized their mouthbrooder host species through lateral transfers (i.e. host switch), as suggested Pouyaud et al. [26]. Our study was limited to Senegal, but we cannot rule out that the host range of generalist *Cichlidogyrus* species infecting two cichlid groups with different reproductive behavioral strategies spans a broader geographical area (i.e. all cichlid species living in Africa). Concerning the generalist *Cichlidogyrus* species reported in our study, prevalence and abundance of *C. halli* 1 and *Cichlidogyrus* sp. 2 were higher in *Sarotherodon galilaeus* than in *Tilapia guineensis*, and higher prevalence and abundance were observed for *C. tilapiae* in *Oreochromis niloticus* than in *Sarotherodon galilaeus*, *Tilapia guineensis* and *Hemichromis fasciatus*. This suggests that generalist monogeneans display a level of host preference, i.e. a generalist selects preferentially one host species within its host range, such as a “common” host species compared to “additional” host species [35]. This supports the hypothesis of a mouthbrooder origin for *Cichlidogyrus* in clade 2 as well as a mouthbrooder origin for the *C. halli* clade.

### Cichlid phylogeny

The cichlid phylogeny based on cytochrome *b* sequences supported three monophyletic groups of African cichlid species: substrate-brooders *Hemichromis*, mouthbrooders *Sarotherodon* and *Oreochromis*, and substrate-brooder *Tilapia*. The third group contains East African cichlid species. This separation of *Sarotherodon* and *Oreochromi*s from *Tilapia* species has been previously reported based on the mitochondrial tRNA^Pro^ gene and the control region sequences [36]. This finding agrees with the hypothesis that the mouthbrooding behavior of *Oreochromis* and *Sarotherodon* genera evolved from a substrate-brooding behavior [36]. Mayer et al. [13] suggested that West African cichlids of the genera *Tylochromis* and *Hemichromis* diverged from the common cichlid stock first and then followed the divergence of *Tilapia* and *Oreochromis*. The separation of mouthbrooders and substrate-brooders is then supported by our phylogenetic analyses based on cytochrome *b*. Following Pouyaud et al. [26], the split between mouthbrooders and substrate-brooders is hypothesized to be linked to the separation of their specific gill parasitofauna. This idea is supported by the observation that *Scutogyrus* parasitizes only mouthbrooders (*Sarotherodon* and *Oreochromis*). Further, *Hemichromis* species possess specific gill monogeneans from the *Onchobdella* genus, not shared by other cichlid species. However, some *Cichlidogyrus* species are able to parasitize both mouthbrooders and substrate-brooder cichlid species (see above). In the present study *Tylochromis intermedius* was found at a basal position relative to the other African cichlids. This basal position of *Tylochromis* among African cichlids supports the observation of Streelman et al. [37] using sequences of the nuclear locus *Tmo-4C4*, and of Zardoya et al. [38] based on microsatellite data. Morphological analyses place *Tylochromis* as a sister group to African tilapiines [12], but this assumption is not supported by molecular studies [39], [40].

### Structural evolution of the haptor

Pouyaud et al. [26] defined four morphological groups within *Cichlidogyrus/Scutogyrus* parasites, “halli”, “scutogyrus”, “tiberianus” and “tilapiae”, using cluster analysis on morphometrical data from haptoral sclerotized parts. Our phylogenetic analyses did not support the monophyly of the “tiberianus” or “tilapiae” groups, because the most diversified clade 5 in our phylogenetic reconstructions included all species classified as “tiberianus” by Pouyaud et al. [26] but also *C. cubitus* classified as “tilapiae”. The mapping of the haptor morphological characters performed in the present study relies on the hypothesis that *C. pouyaudi* diverged early compared to the other *Cichlidogyrus* and *Scutogyrus* species (see above) and thus, the characters of its haptor (two morphologically similar pairs of anchors, all pairs of small marginal hooks, a dorsal bar with small auricles and a ventral bar without membranous extension) are considered to form the ancestral *Cichlidogyrus* haptor type. Moreover, following previous studies on cichlid phylogenies (see above) and Pouyaud et al. [26], we expected to find derived structural characters of haptor in *Cichlidogyrus* from *Tilapia*, *Oreochromis* and *Sarotherodon* compared to *Cichlidogyrus* of *Hemichromis* and *Tylochromis* (because these latter genera display a basal position in the African cichlid phylogeny). The mapping of structural characters in *Cichlidogyrus* and *Scutogyrus* species suggests that the haptor evolved from the simplest type toward the more complex. It also suggests a trend towards a clade-specific morphology with respect to marginal hooks, even if a few changes toward more complicated characters and one reversion to the ancestral state (for *C. cubitus*) were inferred in this analysis. Nevertheless, mapping does not support a different evolution of structural parts of the haptor in mouthbrooder and substrate-brooder cichlids and thus, it does not suggest any morphological adaptation of *Cichlidogyrus* species to the cichlids displaying different reproductive strategies. However, *Cichlidogyrus/Scutogyrus* species of only six cichlid fish species were considered here, and further studies (e.g. taking into account parasites from congeneric *Tilapia* or *Sarotherodon* host species) are needed to confirm these hypotheses. Pouyaud et al. [26] compared two dendrograms, based on morphological data from sclerotized parts of respectively the haptor and the reproductive organ in *Cichlidogyrus/Scutogyrus* parasites, with a phylogeny reconstructed from ribosomal DNA sequences. The two dendrograms were different and only the dendrogram computed from haptor data was congruent with the phylogenetic tree. This suggests that the morphology of the haptor is more suitable for inferring phylogenetic relationships than the morphology of reproductive organs, maybe due to a faster rate of evolutionary change in the morphology of reproductive structures.

### Cophylogenetic analysis of *Cichlidogyrus*/*Scutogyrus* parasites and their cichlid hosts

Because monogeneans are parasites with a direct life cycle and are highly host specific, they have long been considered to cospeciate with their hosts [15], [16], [41]. Recent studies have shown that to the contrary, monogeneans rarely display any significant cospeciation signal with their hosts, and that host-switching and duplication were thought to be important evolutionary events in parasite diversification, e.g. in *Lamellodiscus* [20], *Gyrodactylus* [22], [24], *Polystoma* [25], and *Dactylogyrus* [21], [35]. In the latter studies, *Dactylogyrus* diversification was explained in a large part by intrahost speciation (parasite duplication). All these studies suggest that the high host specificity of monogeneans is not linked to cospeciation. In the present study, distance-based analysis suggests that the global cophylogenetic structure in the *Cichlidogyrus*/*Scutogyrus*-cichlid system is significant. Tree-based analyses, however, indicate that this global structure is not significant, unless if the cost of host-switching is high. That supports previous hypotheses that host-switching, followed by speciation which results in the maintenance of high host specificity, is an important component of monogenean diversification. In all reconstructions, the number of duplications is high, which is also coherent with previously published hypotheses on monogenean evolution. Duplication is then suggested to be the main coevolutionary event explaining the diversification of gill monogeneans living on West African cichlid fish. This has been observed in other gill monogeneans-freshwater fish systems such as *Dactylogyrus*-Cyprinidae [21] and *Thaparocleidus*-Pangasidae (our unpublished data). The fact that global fit is significant with distance-based analysis only suggests that parasites switch to not too distantly related hosts (but not necessarily sister-species). This is supported by the fact that only few individual associations (related to putative cospeciation events, see Legendre et al. [42] and Desdevises et al. [20]) significantly explain this global fit. This confirms the opportunistic behavior and the evolutionary plasticity of monogeneans, which can certainly easily duplicate on hosts, switch hosts and speciate on their new host species, then diversifying at a high rate and maintaining their tremendous diversity.

## Materials and Methods

### Parasite sampling and identification

A total of 28 parasite species belonging to four dactylogyridean genera (*Cichlidogyrus*, *Onchobdella*, *Scutogyrus* and *Enterogyrus*) were collected from the gills and stomachs of cichlid species (Table 2) during field studies in the Niokolo Koba National Park, (Senegal, Africa). Eighty-six cichlid specimens, belonging to six species (*Hemichromis fasciatus*, *Hemichromis letourneuxi*, *Oreochromis niloticus*, *Sarotherodon galilaeus*, *Tilapia guineensis* and *Tylochromis intermedius*), were caught in the Gambie River (Gue de Damantan: 13°1′37″N, 13°11′33″W; Simenti: 13°0′50″N, 13°10′24″W; Camp de Lion: 13°0′53″N, 13°8′41″W), in the Niokolo River (Passage Koba: 13°2′21″N, 13°6′5′W; Pont Suspendu: 13°0′54″N, 13°7′55″W), in the Mare de Simenti (13°1′4″N, 13°10′33″W) and Mare de Wouring (13°7′56″N, 13°11′9″W) and used for this study. *Sarotherodon galilaeus* individuals from Ivory Coast were also investigated for *Scutogyrus* species (because these parasites occur on *S. galilaeus* in Senegal) but none were found during this study.

All cichlids sampled were examined using standard parasitological methodology described in Ergens and Lom [43]. Monogeneans were removed from the gills of freshly killed fish, placed in a drop of water on slides covered by a coverslip, and identified using a light microscope equipped with phase contrast and digital image analysis (Micro Image 4.0 for Windows, Olympus Optical Co., Hamburg, Germany). Parasite determination was performed according to the morphology and size of the sclerotized parts of the haptor (dorsal and ventral anchors, dorsal and ventral transverse bars, marginal hooks) and the reproductive organs (vagina and copulatory organ) following original descriptions [33], [34], [44]–[61]. Parasite specimens were individually preserved in 95% ethanol before DNA extraction. Some specimens from each species were fixed on slides in a mixture of glycerine and ammonium picrate [62].

### Molecular analyses of parasites

Parasites were removed from ethanol and dried, and genomic DNA was extracted using DNeasy™ Tissue Kit (QIAGEN) following the manufacturer's instructions. The LSU rDNA region was amplified using C1 and D2 primers [63]. The amplification reaction was performed using 2 units of *Taq* polymerase (Fermentas), 1x PCR buffer, 1.50 mM MgCl_2_, 0.2 mM of dNTP, 0.50 µM of each primer, 0.1 mg/ml BSA and an aliquot of 30 ng of genomic DNA in a total volume of 30 µl. The polymerase chain reaction (PCR) was carried out using the following steps: 2 min at 94°C followed by 39 cycles of 20 sec at 94°C, 30 sec at 56°C and 1 min 30 sec at 72°C, and then 10 min of final elongation at 72°C. The partial SSU rDNA region and the entire ITS1 were amplified in one round using S1 and IR8 primers [64]. The amplification reaction was performed using 1.5 units of *Taq* polymerase (Fermentas), 1x PCR buffer, 1.50 mM MgCl_2_, 0.2 mM of dNTP, 0.8 µM of each primer, 0.1 mg/ml BSA and an aliquot of 30 ng of genomic DNA in a total volume of 30 µl. PCR was carried out in the Mastercycler ep gradient S (Eppendorf) with the following steps: 2 min at 94°C followed by 39 cycles of 1 min at 94°C, 1 min at 53°C and 1 min 30 sec at 72°C, and 10 min of final elongation at 72°C.

The PCR products were electrophoresed on a 1% agarose gel and then purified by either Wizard® SV Gel and PCR Clean-Up System (PROMEGA) or QIAquick PCR Purification Kit (QIAGEN). Sequencing was performed on an ABI 3130 Genetic Analyzer (Applied Biosystems) using Big Dye Terminator Cycle Sequencing kit version 3.1 (Applied Biosystems). Sequences were analyzed using Sequencher software (Gene Codes Corp) and deposited in EMBL under Accession numbers (see Table 2).

### Phylogenetic analyses of parasite species

DNA sequences were aligned using Clustal W multiple alignment [65] in BioEdit v. 7.0.9 [66]. Gaps and ambiguously aligned regions were removed using GBlocks [67] with the less stringent parameters available in the software. We applied the following criteria “Allow smaller final blocks”, “Allow gap positions within the final blocks” and “Allow less strict flanking positions”. First, phylogenetic analyses using LSU rDNA sequences including *Cichlidogyrus* and *Scutogyrus* as ingroup and *Enterogyrus*, *Onchobdella* and *Protogyrodactylus* as outgroup (following Mendlová et al. [31]) were performed. Next, phylogenetic analyses using partial SSU rDNA and ITS1 sequences of *Scutogyrus* and *Cichlidogyrus* species were performed. The list of parasite species used in the LSU rDNA and/or SSU rDNA and ITS1 alignments is shown in Table 2.

Phylogenetic analyses based on minimum evolution (ME), maximum parsimony (MP) and maximum likelihood (ML) were performed in PAUP*4b10 [68]. Bayesian inference of phylogeny (BI) was computed using MrBayes 3.1.2 [69]. MP analyses were performed using a heuristic search using 10 random searches with a stepwise random addition sequence running on unweighted informative characters and TBR branch swapping. ModelTest [70] was applied to select the most appropriate substitution model of nucleotide evolution for each data set using hierarchical likelihood ratio tests (hLRTs), to be applied in ME, ML (also using heuristic search and TBR) and BI tree reconstructions. ME analysis [71] was performed using heuristic search with a distance optimality criterion. Support for internal nodes were estimated using a bootstrap resampling procedure [72] with 1000 replicates for MP and ME and 100 replicates for ML. Bayesian inference analyses were performed using four Monte Carlo Markov chains running on 3,000,000 generations for LSU rDNA and 1,000,000 for the SSU rDNA and ITS1 data set, with trees being sampled every 100 generations. The “burn-in” asymptote was estimated by plotting the number of generations against the log likelihood scores for the saved trees, and all the trees before stationarity were discarded. In resulting phylogenetic trees, clade support indicated by bootstrap values/posterior probabilities was considered as follows: weak support 50–63%/0.5–0.69, moderate support 64–75%/0.7–0.84, good support 76–88%/0.85–0.94 and strong support 89–100%/0.95–1.00 [73].

### Host phylogeny

The phylogeny of cichlid fishes was previously investigated using mitochondrial DNA sequences [9], [74], [75], nuclear DNA sequences [13], [39], [76] and microsatellite data [37]. However, no previously published study included all the cichlid species investigated in the present study.

Fin clips from cichlid species were preserved in 95% ethanol before DNA extraction. Mitochondrial DNA was isolated with DNeasy Blood and Tissue Kit (QIAGEN) following the manufacturer's instructions. The partial region of cytochrome *b* (434 bp) of *Tilapia guineensis*, *Hemichromis fasciatus*, *H. letourneuxi*, *Oreochromis niloticus* and *Sarotherodon galilaeus* was amplified using forward primer L14725 (5′-CGAAGCTTGATATGAAAAACCATCGTTG-3′) designed by Farias et al. [14] and reverse primer Cichlidae_cytb_1R (5′-WRACKGYAGCVCCTCAGAATGAYA-3′) designed in this study. The partial region of cytochrome *b* (452 bp) of *Tylochromis intermedius* was amplified using forward primer (5′-TTTTACCAGGACTCTAACCAGGA-3′) and reverse primer (5′-GCYCCTCARAATGATATTTGTCC-3′), both of them designed in this study. The PCR reaction mixture consisted of 1.5 units of *Taq* polymerase (Fermentas), 1x PCR buffer, 2.50 mM MgCl_2_, 0.2 mM of dNTP, 0.3 µM of each primer and an aliquot of 30 ng of genomic DNA in a total volume of 30 µl. The PCR was carried out in the Mastercycler ep gradient S (Eppendorf) with the following steps: 4 min at 95°C followed by 30 cycles of 30 sec at 95°C, 30 sec at 50°C and 45 sec at 72°C, and 10 min of final elongation at 72°C. Electrophoresis was performed on 1% agarose gel and the PCR product was purified by Wizard® SV Gel and PCR Clean-Up System (PROMEGA) or QIAquick PCR Purification Kit (QIAGEN). Sequencing was performed on an ABI 3130 Genetic Analyzer (Applied Biosystems) using Big Dye Terminator Cycle Sequencing kit version 3.1 (Applied Biosystems). The sequences of cytochrome *b* were deposited in EMBL under Accession numbers (Table 4). DNA sequences were aligned using Clustal W multiple alignment [65] in BioEdit v. 7.0.9 [66].

Phylogenetic analyses were based on partial cytochrome *b* sequences including 24 fish species as ingroup (i.e. Cichlidae of Africa, Madagascar and South America), and *Cymatogaster*, *Etheostoma* and *Halichoeres* as outgroup (Cichlidae of North America and Western Pacific selected following Farias et al. [14]). Because these coding sequences were highly conserved and of the same size (342 positions after aligning the cytochrome *b* sequences of our cichlid species with the sequences available in GenBank, see Results section), no insertion or deletion event nor trimming was needed to improve the alignment, that was carefully checked visually. The list of fish species and accession numbers for cytochrome *b* sequences are shown in Table 4. Bayesian inference analyses were performed using four Monte Carlo Markov chains running on 2,000,000 generations with trees being sampled every 100 generations. Cytochrome *b* DNA sequences were considered with an evolutionary model designed for coding sequences taking the genetic code into account [77], [78], as well as a codon partition scheme considering independently each position within the codon [79]. The low variability within the alignment precluded the use of translated sequences in phylogenetic reconstructions.

### Cophylogenetic analyses

Two methods of coevolutionary analyses were used: a distance-based method called ParaFit [42] implemented in CopyCat [80] and a tree-based method implemented in Jane 3.0 [81]. Note that Jane 3.0 was designed for a good handling of widespread parasites (i.e. using multiple hosts), as it is the case in this study, and that ParaFit was tested for such situation [42], for which it was shown to be efficient. A useful review of existing methods for cophylogenetic studies is given in Light and Hafner [82]. A tanglegram representing the host-parasite associations was reconstructed using TreeMap 1.0 [83].

Distance-based methods focus only on the fit between host and parasite distances and do not test for the presence of any coevolutionary events. These methods use distance matrices and host associations to determine if hosts and parasites are randomly associated. An advantage of this is that they do not require fully resolved phylogenetic trees and can account for parasites associated with multiple hosts. In this study, patristic distances were calculated in CopyCat for each host and parasite phylogeny. The global fit between trees is computed and tested by randomizing individual host-parasite associations (links). ParaFit was also used to test whether a particular host-parasite link contributed to this global fit. Tests of significance were performed using 999 permutations.

Tree-based methods use topologies and branch lengths to assess the fit between host and parasite phylogenies. These methods attempt to reconstruct the shared evolutionary history between hosts and their parasites with the smallest “cost” or smallest number of hypothesized historical events. A disadvantage of tree-based methods is that they require fully resolved phylogenies, and then do not account for phylogenetic uncertainty. Some of them, as TreeMap 1.0, do not appropriately account for parasites associated with multiple hosts in certain cases and therefore may underestimate host switching [83], [84]. To overcome this problem, we conducted analyses with Jane [81], using different event costs schemes. In addition to the four types of coevolutionary events classically used in such studies i.e. cospeciation, duplication (parasite speciation without host speciation), host switching, and sorting, Jane uses a fifth type named “failure to diverge”, referring to the instances when a host speciation is not followed by parasite speciation, which remains as the same species on the newly produced host species. Each type of event is attributed a cost and the algorithm searches the reconstruction with the lowest global cost. In our study, we used the fully resolved minimum evolution parasite tree inferred from the analysis of combined SSU rDNA and ITS1 data. Seven models with different event costs scheme were used for the cophylogenetic analyses performed in Jane (see Table 3), using 500 generations and a population size of 50 as parameters of the genetic algorithm. Statistical tests were computed using 999 randomizations with random parasite trees.

### Mapping of the morphology of attachment apparatus onto the parasite phylogenetic tree

We investigated whether morphological evolution, i.e. evolution of the attachment apparatus, is linked to the parasite phylogeny. Categorical coding was used for character states, which were unordered because no relevant hypotheses could be applied for character polarization. Morphological characters of the haptor were mapped onto the fully resolved minimum evolution parasite tree inferred from the analysis of combined SSU rDNA and ITS1 data using MacClade version 4.0.1 with Farris optimization [85]. The following morphological characters were evaluated: shape of the anchors, shape of marginal hooks, shape of the ventral transverse bar, and shape of the dorsal transverse bar (see Table 5 for character states). The morphological characters of the sclerotized parts of the haptor and character states are shown on Figure 6. Two character states were defined for anchors: similar shape of both pairs of anchors and different shape of the first (ventral) and second pair (dorsal) of anchors. Four character states were defined for marginal hooks: first pair of large hooks and 3rd to 7th pair of small hooks, all pairs of small hooks, first pair of small hooks and 3rd to 7th pair of large hooks, and all pairs of large hooks. The morphology of the second pair of hooks was not considered in this analysis because of its small size in all parasite species. For the ventral bar, four morphological types were defined: bar with membranous extension, bar without membranous extension, massive bar with membranous extension, and arched bar supporting one large, thin, oval plate marked by fan-shaped median thickenings. Finally, three character states were defined for the dorsal bar: bar with two well developed auricles on the ventral face of the bar, bar with two very long auricles and lateral outgrowths, and bar with two small auricles on the anterior face of the bar.
